# Free Fatty Acids Profiles Are Related to Gut Microbiota Signatures and Short-Chain Fatty Acids

**DOI:** 10.3389/fimmu.2017.00823

**Published:** 2017-07-24

**Authors:** Javier Rodríguez-Carrio, Nuria Salazar, Abelardo Margolles, Sonia González, Miguel Gueimonde, Clara G. de los Reyes-Gavilán, Ana Suárez

**Affiliations:** ^1^Department of Microbiology and Biochemistry of Dairy Products, Instituto de Productos Lácteos de Asturias (IPLA-CSIC), Villaviciosa, Asturias, Spain; ^2^Area of Physiology, Department of Functional Biology, University of Oviedo, Oviedo, Asturias, Spain; ^3^Area of Immunology, Department of Functional Biology, University of Oviedo, Oviedo, Asturias, Spain

**Keywords:** free fatty acids, microbiota, *Akkermansia*, short-chain fatty acids, serum lipids, subclinical metabolic alterations

## Abstract

A growing body of evidence highlights the relevance of free fatty acids (FFA) for human health, and their role in the cross talk between the metabolic status and immune system. Altered serum FFA profiles are related to several metabolic conditions, although the underlying mechanisms remain unclear. Recent studies have highlighted the link between gut microbiota and host metabolism. However, although most of the studies have focused on different clinical conditions, evidence on the role of these mediators in healthy populations is lacking. Therefore, we have addressed the analysis of the relationship among gut microbial populations, short-chain fatty acid (SCFA) production, FFA levels, and immune mediators (IFNγ, IL-6, and MCP-1) in 101 human adults from the general Spanish population. Levels of selected microbial groups, representing the major phylogenetic types present in the human intestinal microbiota, were determined by quantitative PCR. Our results showed that the intestinal abundance of *Akkermansia* was the main predictor of total FFA serum levels, displaying a negative association with total FFA and the pro-inflammatory cytokine IL-6. Similarly, an altered FFA profile, identified by cluster analysis, was related to imbalanced levels of *Akkermansia* and *Lactobacillus* as well as increased fecal SCFA, enhanced IL-6 serum levels, and higher prevalence of subclinical metabolic alterations. Although no differences in nutritional intakes were observed, divergent patterns in the associations between nutrient intakes with intestinal microbial populations and SCFA were denoted. Overall, these findings provide new insights on the gut microbiota–host lipid metabolism axis and its potential relevance for human health, where FFA and SCFA seem to play an important role.

## Introduction

Free fatty acids (FFA) are lipid species released from the adipose tissue and several cell types upon lipolysis. Apart from their classical roles in energy supply or as structural components, FFA are emerging as active players of a number of biological processes. FFA can affect gene expression of macrophages (1), adipocytes (2), or endothelial cells (3). In addition, FFA can modulate the production of chemokines and cytokines (3–5), the expression of genes coding for adhesion molecules (6, 7) and they give rise to pro-inflammatory and inflammation pro-resolving lipid-derived species (8).

Therefore, a growing body of evidence emphasizes a role for FFA as common mediators between metabolic conditions and the immune system. FFA have been proposed as a mechanistic explanation for the relationship among obesity, inflammation, altered glucose homeostasis, and cardiovascular disease (9). Similarly, some FFA have been reported as regulators of systemic metabolism homeostasis in mice (10). Importantly, quantitative and qualitative differences among the effects of individual FFA have been revealed (11). Thus, not only increased levels but also an altered FFA pool composition may be associated with the risk of developing a range of disorders in which the immune system plays a role (12–17). However, the underlying causes of the altered FFA pool composition remain unknown.

Compelling evidence from recent years has shed some light on the effects that gut microbiota can exert on the host health. Alterations in the composition of the intestinal microbiota have been related with the development of metabolic disorders both in mice and in human studies (18), whereas some dietary factors are also known to modulate these microbial communities (19). Recent studies indicate that the gut microbiota is also involved in the host energy metabolism by regulating the absorption of nutrients, local production of hormones and immune mediators, fat storage, and gut permeability (20–22). However, to what extent host lipid metabolism is associated with the intestinal microbial populations, and whether the gut microbiota could be related to FFA levels, remains largely unknown. Short-chain fatty acids (SCFA) are produced in the gut by the metabolic activity of the intestinal microbiota as catabolic end-products from the fermentation of undigested dietary components, mainly complex carbohydrates. These compounds are pivotal in the interactions between the host and intestinal microbial populations (23), but their actual links with the metabolism of fatty acids in humans are controversial. In addition, since most studies have been performed with relatively low sample sizes and mainly with *a priori* established conditions, evidence for their potential relevance in healthy adults is lacking in the literature. However, due to their pivotal role in shaping the host metabolism, it is tempting to speculate that the intestinal microbiota could be related to FFA levels in healthy populations.

Based on these lines of evidence, we hypothesize that alterations in the composition and metabolic activity of the gut microbiota may be associated with altered levels of FFA, which can be in turn be related with some mediators of inflammation. With the aim to gain insight into the joint impact of these players in human health, we have studied the relationships among selected microbial populations, fecal SCFA, serum FFA levels, and composition, as well as inflammatory mediators. In this way, the main objective of the present study was to evaluate whether specific gut microbial signatures can be related to altered levels of FFA species in adults from the general population.

## Materials and Methods

### Ethical Approval

Ethical approval for this study was obtained from the Institutional Review Board (Comité de Ética de Investigación Clínica del Principado de Asturias) in compliance with the Declaration of Helsinki. All participants were informed and gave a signed informed consent prior their inclusion in the study.

### Subjects

A group of 101 individuals was recruited from the general population in the Asturias region, northern Spain. Exclusion criteria were the diagnosis of chronic or immune-mediated diseases, recent infections, cancer, or altered metabolic conditions, as well as the current usage (within the previous 6 months) of immunomodulatory drugs, metabolic agents, probiotics, or antibiotics. Demographical parameters of the analyzed population are summarized in Table 1.

**Table 1:** Description of the study population.

	Study population (*n* = 101)
**Demographical parameters**	
Age, mean (range)	49.41 (19–72)
Gender (F/M)	66/35
BMI	26.76 ± 4.39
**Biochemical parameters (mg/dl)**	
Glucose	96.49 ± 12.20
Total cholesterol	208.66 ± 41.64
HDL cholesterol	56.70 ± 12.84
LDL cholesterol	130.41 ± 35.80
Triglycerides	107.53 ± 62.78
**Microbial populations (log cells/g)**	
*Akkermansia*	5.84 ± 1.80
*Bacteroides* group	9.15 ± 1.16
*Bifidobacterium*	8.26 ± 0.91
*Clostridium* cluster XIVa	8.52 ± 1.41
*Lactobacillus* group	6.14 ± 1.04
*Faecalibacterium*	8.05 ± 1.29
**Main short-chain fatty acid (SCFA) (mM)**	
Acetate	40.46 ± 19.20
Propionate	15.04 ± 8.34
Butyrate	10.61 ± 7.19
Total SCFA	66.42 ± 31.06
**Free fatty acids (FFA) (μg/ml)**	
Palmitic (16:0)	28.34 (12.04)
Stearic (18:0)	28.35 (9.98)
Palmitoleic (16:1w7)	0.80 (1.20)
Oleic (18:1w9)	27.38 (20.12)
Linoleic (18:2w6)	7.90 (7.11)
γ-Linoleic (18:3w6)	0.08 (0.08)
AA (20:4w6)	2.02 (1.49)
Linolenic (18:3w3)	0.18 (0.16)
Eicosapentaenoic (20:5w3)	0.09 (0.16)
Docosahexaenoic acid (22:6w3)	1.31 (1.59)
SFA	58.28 (19.08)
MUFA	28.15 (20.48)
w6-PUFA	10.59 (8.21)
w3-PUFA	1.67 (1.74)
Total FFA (mM)	0.31 (0.17)
**Cytokine serum levels (pg/ml)**	
IL-6	442.79 (949.99)
MCP-1	267.30 (127.83)
IFNγ	2.05 (4.70)

*Variables are summarized as median (interquartile range), mean ± SD, or *n* (%), as appropriated, unless otherwise stated*.

Subjects were asked to participate in this study and, upon acceptance, an overnight fast blood sample was drawn by venipuncture in tubes without anticoagulant. After blood clotting, serum was collected and stored at −80°C until further analyses. In addition, basic serum biochemical analyses were performed by standardized procedures. Subjects were considered to exhibit a subclinical metabolic alteration if they meet any of the following criteria: fasting glucose levels higher than 100 mg/dl or that of triglycerides over 150 mg/dl. These objectives cut offs were obtained from clinical national guidelines.

### Total FFA Assessment

Total FFA serum levels were quantified by means of a colorimetric assay using a commercial kit (NEFA kit half-microtest, Roche Life Sciences, Penzberg, Germany) following the protocol from the manufacturer. Final absorbance was measured at 546 nm, and detection limit was 0.02 mM.

### Individual FFA Quantification

Free fatty acids were analyzed after a methyl-*tert*-butylether (MTBE)-based extraction protocol as previously described (24) with slight modifications. Briefly, 100 µl serum samples were spiked with 5 µl of internal standard (600 ppm heptadecanoic acid). Then, protein precipitation was performed by adding 200 µl methanol chromasolv grade (Sigma Aldrich, MO, USA), and tubes were vortexed for 30 s. Then, 1,200 µl MTBE chromasolv grade (Sigma) was added, vortexed, and an incubation in an ultrasound water bath at 15°C was performed for 30 min. Finally, organic phase was separated after the addition of 200 µl milliQ water and a centrifugation step for 7 min at 5,000 rpm (15°C). After collecting the upper layer, the extraction protocol was repeated once with 100 µl MetOH, 500 µl MTBE, and 100 µl milliQ H_2_O.

Lipid extracts were dried in a miVac centrifugal evaporator (Genevac Ltd., UK) and redissolved in 100 µl of water:acetonitrile 38:62.

For the determination of the fatty acids, a Dionex Ultimate 3000 HPLC system (Thermo Scientific, Bremen, Germany), consisting of a high pressure binary pump, an autosampler and a column oven, was used. The column was a Zorbax Eclipse Plus C18, 50 mm × 2.1 mm, 1.8 μm from Agilent. Mobile phases A and B were water and acetonitrile, respectively, both containing 0.1% of formic acid. Fatty acids separation was carried out by the following gradient program: 62% B (held for 4.5 min) followed by a linear increase up to 100% B in 10 min (held for 1 min). The column temperature was set at 45°C and the injection volume was 2 µl.

Mass detection was performed using a Bruker Impact II q-ToF mass spectrometer with electrospray ionization, operated in the negative mode. The settings of the mass spectrometer were as follows: spray voltage, 4.5 kV; drying gas flow 12 l/min; drying gas temperature 250°C; nebulizer pressure 44°psi.

For quantitation, calibration curves for each compound were prepared by proper dissolution of the pure standards in methanol to encompass the expected concentration of the analytes in the sample. The calibration ranges were as follows: 0.4–12.5 µg/ml for eicosapentaenoic (EPA) and γ-linolenic; 1.2–37.5 µg/ml for docosahexaenoic acid (DHA) and linolenic; 2.3–75 µg/ml for arachidonic (AA) and palmitoleic; 3.9–125 µg/ml for linoleic; and 7.8–250 µg/ml for oleic, palmitic, and stearic. A good linearity was observed in all cases (*r*^2^ > 0.994). Heptadecanoic acid, added at a level of 30 µg/ml, was used as internal standard to compensate for possible biases during the sample preparation step.

### Quantification of Cytokines

Serum levels of IFNγ, MCP-1, and IL-6 were determined by immunoassays using commercial kits (IFNγ OptEIA kit) from BD Biosciences (NJ, USA) and MCP-1 and IL-6 mini-EDK kits from Peprotech (NJ, USA), following manufacturer’s instructions. Detection limits were 0.58, 8, and 5.8 pg/ml for IFNγ, MCP-1, and IL-6, respectively.

### Nutritional Assessments

Dietary intake was assessed by means of an annual semi quantitative validated food frequency questionnaire including 160 items (25). Trained dieticians asked about cooking practices, number and amount of ingredients used in each recipe, as well as enquiring about menu preparation (e.g., type of oil used and type of milk) and other relevant information to the study. During an interview, subjects were asked item by item whether they usually ate each food and, if so, how much they usually ate. For this purpose, three different serving sizes of each cooked food were presented in pictures to the participants so that they could choose from up to seven serving sizes (from “less than the small one” to “more than the large one”). For some of the foods consumed, amounts were recorded in household units, by volume, or by measuring with a ruler. Methodological issues concerning dietary assessment have been detailed elsewhere (25). Food intake was analyzed for energy, macronutrients, and total fiber content by using the nutrient Food Composition Tables developed by the Centro de Enseñanza Superior de Nutrición Humana y Dietética (26).

### Anthropometric Measures

Height was measured using a stadiometer with an accuracy of ±1 mm (Año-Sayol, Barcelona, Spain). The subjects stood barefoot, in an upright position and with the head positioned in the Frankfort horizontal plane. Weight was measured on a scale with an accuracy of ±100 g (Seca, Hamburg, Germany). Quetelet index was calculated using the formula: weight (kg)/height (m^2^).

### Analysis of Fecal Microbiota

Fecal samples from individuals participating in the study were collected at home in sterile containers, kept at 4°C in the domestic refrigerator (maximum 2–3 h from deposition) and frozen at −80°C until analyses just right on arrival to the laboratory, as previously reported (27, 28). One gram of fecal sample was employed for DNA extraction by using the QIAamp DNA stool mini kit (Qiagen, Hilden, Germany) as previously described (28). Quantification of bacterial groups (*Akkermansia, Bacteroides* group, *Bifidobacterium, Clostridium* cluster XIVa, *Lactobacillus* group, and *Faecalibacterium*) in feces was performed with a 7500 Fast Real Time PCR System (Applied Biosystems, Foster City, CA, USA) using SYBR Green PCR Master Mix (Applied Biosystems) as previously described (29–31). These microbial groups include the main representatives of the human intestinal microbiota, which all together represent over 95% of the total bacteria in the human intestine (32, 33). For qPCR analysis, 1 µl of template fecal DNA (~5 ng) and 0.2 µM of each primer were added to the reaction mixture (25 µl). PCR cycling was as follows: an initial cycle of 95°C 10 min, 40 cycles of 95°C 15 s, and 1 min at the appropriate primer temperature. We compared the *C*_t_ values obtained from a standard curve constructed as previously indicated (30) to estimate the number of cells. Standard cultures, primers, and annealing temperatures used for qPCR were the same as those recently reported (31).

### Analysis of Short-Chain Fatty Acids in Fecal Samples

Analysis of short chain fatty acids (SCFA) was performed by gas chromatography to determine the concentrations of acetate, propionate, and butyrate. One gram of fecal samples was weighed, diluted 1:10 in sterile PBS, and homogenized in a LabBlender 400 stomacher (Seward Medical, London, UK) at full speed for 4 min. Supernatants were then obtained by centrifugation (10,000 *g*, 30 min, 4°C), filtered through 0.2-µm filters, mixed with 1/10 of ethyl butyric (2 mg/ml) as an internal standard, and stored at −80°C until analysis. A gas chromatograph 6890N (Agilent Technologies Inc., Palo Alto, CA, USA) connected to a mass spectrometry (MS) 5973N detector (Agilent Technologies) and to a flame ionization detector was used for identification and quantification of SCFA.

Data were collected using the Enhanced ChemStation G1701DA software (Agilent). Samples (1 µl) were directly injected into the gas chromatograph equipped with an HP-Innowax capillary column (60-m length by 0.25-mm internal diameter, with a 0.25 µm film thickness; Agilent) using He as gas carrier and a constant flow rate of 1.5 ml/min. The temperature of the injector was kept at 220°C, and the split ratio was 50:1. Chromatographic conditions were as follows: initial oven temperature of 120°C, 5°C/min up to 180°C, 1 min at 180°C, and a ramp of 20°C/min up to 220°C to clean the column. In the MS detector, the electron impact energy was set at 70 eV. The data collected were in the range of 25–250 atomic mass units (at 3.25 scans/s).

SCFA were identified by comparison of their mass spectra with those held in the HP-Wiley 138 library (Agilent) and by comparison of their retention times with those of the corresponding standards (Sigma Aldrich, St. Louis, MO, USA). The peaks were quantified as relative abundances with respect to the internal standard. The concentration (in mM) of each SCFA was calculated using the linear regression equations (*r*^2^ ≥ 0.99) from the corresponding standard curves obtained with six different concentrations.

### Statistical Analysis

**Figure 1:**
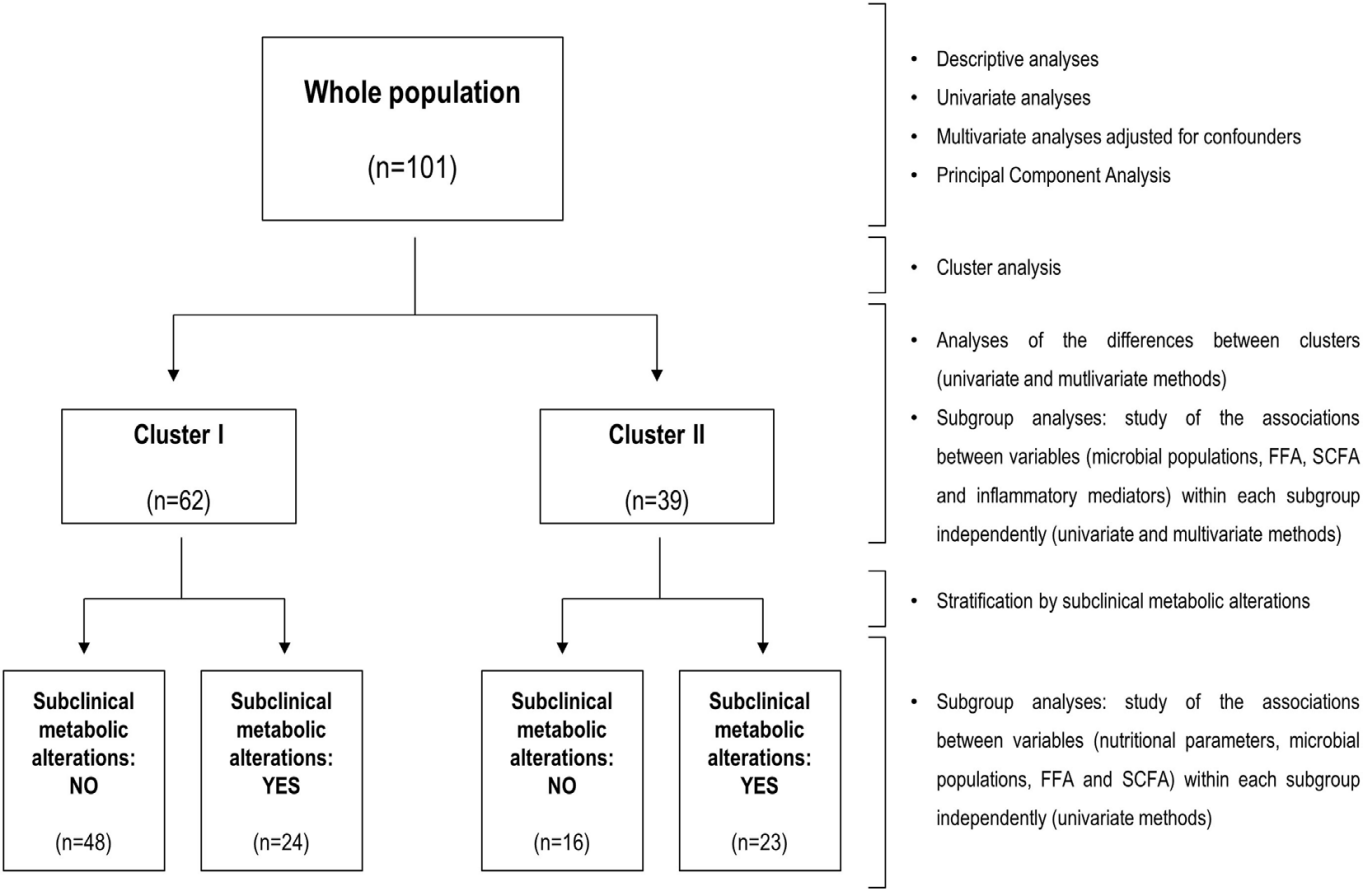
Flow diagram of the statistical analyses. Summary of the workflow followed in the statistical analyses performed in the present study. The flow diagram (left) indicates groups analyzed and sample sizes, whereas statistical methods for analysis and stratification of the study population are indicated at the left side.

The study design was a cross-sectional, one-group, observational analysis. Continuous variables were summarized as median (interquartile range) or mean ± SD, whereas *n* (%) was used for the categorical ones. Mann–Whitney *U* or *t*-tests were used to assess statistical differences, when appropriate. Correlations were analyzed by Spearman’s rank test. After univariate correlation analyses, multiple regression analyses were conducted to assess the strength of the association between these variables including other (continuous) variables as potential confounders. Multiple regression analyses were also performed to identify the main predictors of a candidate independent variable. When coefficients of determination correspond to multiple regression analyses they are indicated as R^2^. For each analysis, dependent and independent variables were indicated and the β coefficient, *B* coefficient with 95% confidence intervals (CIs), and *p*-values were computed. The association between two categorical variables was first studied by χ^2^ tests in univariate models and then was analyzed by multiple logistic regression models to include potential confounders as independent variables. For this analysis, odds ratio (OR) and 95% CI were computed. Principal component analysis (PCA) with Varimax rotation was performed to reduce sample dimensionality and potential collinearity effects. The number of components retained was based on eigenvalues (>1), and loadings greater than 0.5 were used to identify the variables comprising a single component. For cluster analysis, squared Euclidean distances were calculated, and Ward’s Minimum Variance Method was used to identify clusters minimizing the loss of information. R package *heatmap.2* was used to generate heatmaps for visualization purposes. Some statistical analyses were performed independently in each cluster to evaluate whether differences among the studied variables independently arise on each cluster. The statistical approach is summarized in Figure 1. SPSS 19.0, R 3.0.3, and GraphPad Prism 5.0 for Windows were used.

## Results

### FFA and Microbial Populations

**Figure 2:**
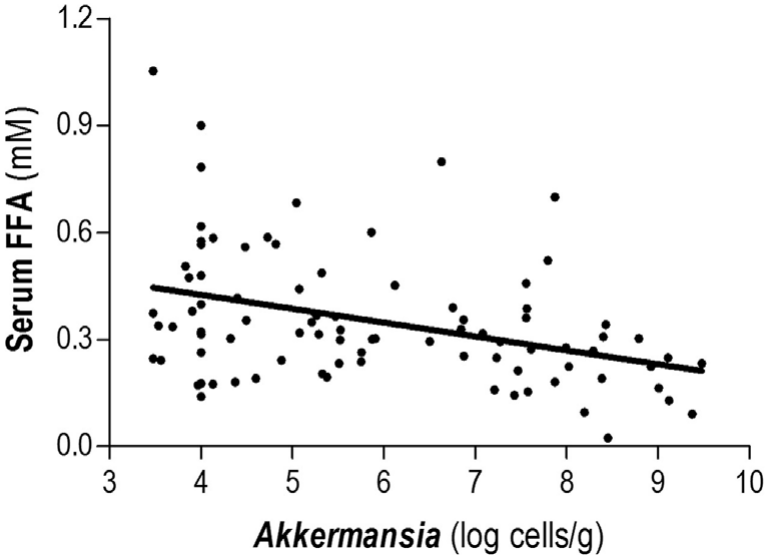
Association between *Akkermansia* and serum total free fatty acids (FFA) levels. Abundance of *Akkermansia* in fecal samples was negatively correlated with the total levels of serum FFA in healthy subjects. Correlation was assessed by Spearman rank’s test.

Total FFA serum levels and fecal microbial groups were analyzed in 101 human adults from the general population (Table 1). High total FFA serum levels were considered as a surrogate marker of impaired lipid metabolism. Then, the associations between FFA levels and intestinal microbial populations were determined by univariate and multivariate analyses adjusted for socio-demographical [age, gender, and body mass index (BMI)] and nutritional parameters (total energy and intakes of carbohydrates, lipids, proteins, and fiber). Interestingly, only *Akkermansia* abundance was correlated with FFA serum levels (*r* = -0.383, *p* < 0.001) (Figure 2). Moreover, this association remained statistically significant in a multiple regression analysis adjusted for age, gender, BMI, microbial groups analyzed, total energy as well as carbohydrates, lipids, and proteins intake as potential confounders (Table 2). Although a slight correlation between total FFA and the serum levels of the pro-inflammatory cytokine IL-6 was observed (*r* = 0.240, *p* = 0.020), no effect was evidenced when IL-6 was introduced in the previous multiple regression analysis.

**Table 2:** *Akkermansia* as predictor of free fatty acids (FFA) serum levels.

	β	*B* [95% CI]	*p*-Value
Age	−0.099	−0.001 [−0.006, 0.003]	0.515
Gender	−0.119	−0.045 [−0.131, 0.042]	0.309
BMI	0.078	0.003 [−0.006, 0.012]	0.463
*Akkermansia*	−0.350	−0.035 [−0.055, −0.014]	**<0.001**
*Bacteroides* group	−0.080	−0.013 [−0.055, 0.029]	0.550
*Bifidobacterium*	−0.041	−0.008 [−0.055, 0.039]	0.742
*Clostridium* cluster XIVa	−0.064	−0.008 [−0.041, 0.025]	0.627
*Lactobacillus* group	0.180	0.031 [−0.013, 0.074]	0.169
*Faecalibacterium*	0.092	0.015 [−0.038, 0.067]	0.584
Total energy	0.141	0.001 [−0.001, 0.002]	0.141
Carbohydrates intake	0.604	0.002 [−0.001, 0.004]	0.160
Lipids intake	0.606	0.004 [−0.001, 0.009]	0.146
Proteins intake	0.173	0.001 [−0.002, 0.005]	0.510

*The associations between FFA serum levels (as dependent variable) and microbial populations were studied by multiple lineal regression analysis including sociodemographical parameters and nutritional intakes as potential confounders (all included as independent variables in the model). β and *B* coefficients [with 95% confidence intervals (CI)] and *p*-values are calculated for each parameter within the same model. *R*^2^ (model) = 0.303. The *p*-value highlighted in bold represent statistically significant differentes*.

Next, we aimed to evaluate whether this finding was due to a general effect on total FFA levels or it could be also associated with individual FFA species. To this end, associations between *Akkermansia* and the levels of individual FFA (γ-linolenic, palmitic, oleic, stearic, linoleic, palmitoleic, linoleic, AA, EPA, and DHA) were assessed. *Akkermansia* abundance was negatively associated with some FFA [stearic (*r* = −0.218, *p* = 0.039), palmitic (*r* = −0.321, *p* = 0.002), oleic (*r* = −0.261, *p* = 0.013), palmitoleic (*r* = −0.297, *p* = 0.004), linoleic (*r* = −0.272, *p* = 0.010), and γ-linolenic (*r* = −0.232, *p* = 0.028)], but no association was observed with EPA, DHA, AA, or linoleic levels. As important differences among FFA exist, individual FFA were grouped according to their chemical structure: saturated (SFA), monounsaturated (MUFA), w3-poly-unsaturated (w3-PUFA), and w6-PUFA. Interestingly, *Akkermansia* exhibited a strong negative association with SFA (*r* = −0.314, *p* = 0.003) as well as with MUFA and w6-PUFA to a lower degree (*r* = −0.266, *p* = 0.011 and *r* = −0.244, *p* = 0.020, respectively), whereas no association was observed for w3-PUFA (*r* = −0.104, *p* = 0.330). Thus, our findings point to a negative association between *Akkermansia* abundance and levels of SFA, MUFA, and w6-PUFA, all of them pro-inflammatory. Since some collinearity was noted among individual FFA levels, a PCA was carried out to avoid potential biases. PCA was then conducted with the serum levels of specific FFA, given that all of them exhibited communalities higher than 0.5, thus supporting the appropriateness of this analysis. The Kaiser–Meyer–Olkin test provided a good adequacy of the data (0.858) as the Bartlett test of sphericity (*p* = 10^−144^) did. PCA identified two main components (explaining 72.4% of the total variance): C1 (including loadings from γ-linolenic, palmitic, oleic, stearic, linoleic, palmitoleic, linoleic, AA; explaining 59.81% of the total variance) and C2 (including EPA and DHA; explaining 13.09% of the total variance). Notably, *Akkermansia* was negatively associated with C1 (*r* = −0.308, *p* = 0.003), thus supporting an opposite relationship between the levels of this microorganism and those of the saturated, monounsaturated, and w6-PUFA retained in this component, whereas no association was found with anti-inflammatory w3-PUFA (C2). These results from the PCA strengthen our previous findings on the separated analysis of FFA according to their chemical structure. Moreover, since PUFA are present in lower levels, and taking into account that nine FFA loaded on C1 whereas only two did in C2, this association was studied by regression analyses including total FFA serum levels as confounder to rule out the possibility that decreased FFA abundance may bias the analysis. Interestingly, *Akkermansia* levels remained associated with C1 after performing this adjustment (β [95% CI], *p*: −0.286 [−1.999, −0.315], *p* = 0.008), whereas no association was observed with C2 (−0.052 [−0.152, −0.092], *p* = 0.625), thereby confirming our previous findings. In addition, the analysis of inflammatory mediators showed that *Akkermansia* was negatively associated with IL-6 serum levels (*r* = −0.233, *p* = 0.032), whereas IL-6 levels and C1 were in turn associated (*r* = 0.220, *p* = 0.032). No associations were found for *Akkermansia* with IFNγ (*r* = 0.056, *p* = 0.604) or MCP-1 (*r* = −0.028, *p* = 0.802) serum levels.

Therefore, all these results suggest a relationship between intestinal *Akkermansia* and serum FFA levels in humans. Moreover, *Akkermansia* was negatively associated with specific FFA species, mainly those saturated and/or with an attributed pro-inflammatory role, such as MUFA and w6-PUFA. This finding is in line with the negative association observed between *Akkermansia* and IL-6 serum levels.

### Associations between FFA Profiles, Microbial Populations, and SCFA

**Figure 3:**
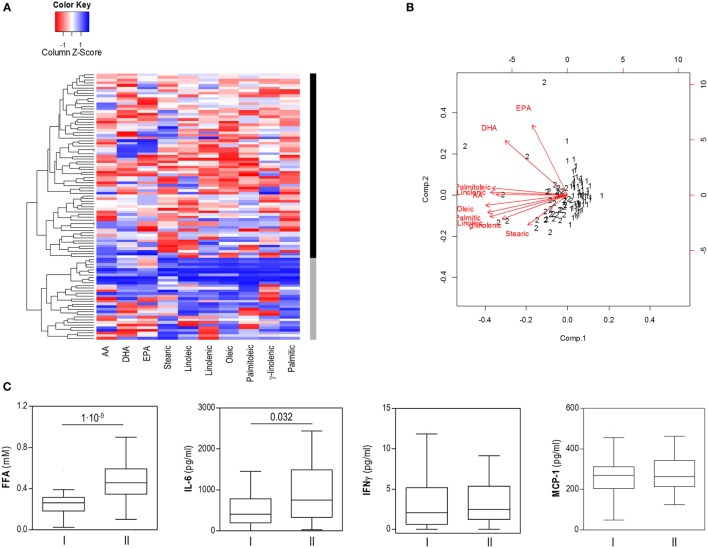
Cluster analysis of free fatty acids (FFA) based on specific FFA serum levels. **(A)** Heatmap showing dendrogram classification for the clusters based on specific FFA serum levels (columns). Colors in the vertical bar at the right of the heatmap identify clusters I (black) and II (gray). Tiles are colored based on serum FFA concentrations, red and blue indicating low or high levels, respectively. **(B)** Biplot obtained in the cluster analysis of specific FFA serum levels. Red arrows represent the vectors showing the associations among the original variables entered and the outcome of the cluster analysis. Numbers represent the final cluster assigned to each subject (1: cluster I, 2: cluster II). Whereas saturated and mainly pro-inflammatory FA were closely related, EPA and DHA were not involved in clusters definition. **(C)** Comparison of serum levels of FFA, IL-6, IFNγ, and MCP-1 between subjects of both clusters. Boxes represent median and interquartile range, whereas whiskers represent minimum and maximum values. Differences were assessed by Mann–Whitney *U* tests.

Our results from the PCA analysis and the differences found in the associations with *Akkermansia* and IL-6 levels point to the existence of different FFA profiles among individuals. To address this hypothesis, an unsupervised cluster analysis was performed for the individual FFA levels. Interestingly, this approach confirmed the existence of two independent groups of individuals based on FFA levels, thereafter referred to as cluster I (*n* = 62) and cluster II (*n* = 39) (Figure 3A).

Although all FFA species were higher in cluster II, mainly saturated and/or pro-inflammatory fatty acids were associated with this group, whereas a strikingly different pattern was observed for EPA and DHA, which seem not to play a role in clusters definition on our analysis (Figure 3B). No significant differences in age, gender or BMI were observed between individuals from clusters I and II (Table 3). Nutrient intakes were then compared between clusters in order to evaluate whether distinct daily intakes could account for the differences observed in FFA clusters. Interestingly, no differences in daily intakes were found (Table 4), hence supporting that additional factors other than demographic and nutritional parameters may underlie the differences found between FFA clusters.

**Table 3:** Demographical parameters according to free fatty acids (FFA) clusters.

	Study population (*n* = 101)	Cluster I (*n* = 62)	Cluster II (*n* = 39)	*p*-Value
**Demographical parameters**				
Age, mean (range)	49.41 (19–72)	50.39 (25–72)	47.78 (19–67)	0.260
Gender (F/M)	66/35	42/20	24/15	0.524
BMI	26.76 ± 4.39	26.67 ± 4.50	26.91 ± 4.27	0.580

*Demographical parameters from the whole population (*n* = 101) and from the study participants classified according to clusters computed from individual FFA levels are shown. Differences between clusters were analyzed by Mann–Whitney *U* or χ^2^ tests. Variables are expressed as mean ± SD, median (interquartile range) or *n*, as appropriate*.

**Table 4:** Nutritional parameters according to free fatty acids (FFA) clusters.

	Study population (*n* = 101)	Cluster I (*n* = 62)	Cluster II (*n* = 39)	*p*-Value (unadjusted)	*p*-Value (adjusted)
**Daily intakes**					
Energy (kcal)	1,993.24 ± 531.62	1,961.40 ± 536.41	2,011.51 ± 515.05	0.660	0.842
Lipids (g)	80.49 ± 27.34	80.64 ± 26.31	78.23 ± 27.77	0.494	0.126
Saturated (g)	26.24 ± 10.00	26.82 ± 10.82	25.04 ± 8.54	0.512	0.109
Monounsaturated (g)	33.83 ± 13.25	35.08 ± 12.82	30.68 ± 11.86	0.100	0.079
Polyunsaturated (g)	13.94 ± 9.06	12.41 ± 6.24	15.91 ± 11.96	0.410	0.138
Proteins (g)	92.15 ± 27.66	93.36 ± 28.79	88.76 ± 25.58	0.754	0.132
Carbohydrates (g)	213.06 ± 69.43	207.73 ± 66.70	226.79 ± 73.77	0.125	0.070
Fiber (g)	22.71 ± 10.65	23.46 ± 11.74	21.75 ± 8.88	0.532	0.249
Soluble (g)	2.75 ± 1.21	2.79 ± 1.16	2.74 ± 1.32	0.551	0.972
Insoluble (g)	14.13 ± 5.88	14.50 ± 6.11	13.78 ± 5.57	0.523	0.545

*Dietary intakes from the whole population (*n* = 101) and from the study participants classified according to clusters computed from individual FFA levels are shown. Differences in daily intakes between clusters were analyzed by Mann–Whitney *U* tests (unadjusted) or linear regression models to adjust for confounders (adjusted). Energy was adjusted by gender and age, whereas the rest of the nutrients were adjusted by gender, age, and energy. Variables are expressed as mean ± SD*.

Notably, important differences in intestinal microbial populations and fecal SCFA levels were observed between clusters (Table 5). Cluster II was characterized by decreased levels of *Akkermansia* and increased *Lactobacillus* group counts as well as higher acetate, propionate and total fecal SCFA concentrations than cluster I. Moreover, increased total FFA [0.48 (0.24) vs 0.26 (0.13) mM] and IL-6 [752.40 (1,155.22) vs 411.77 (636.85) pg/ml], but not IFNγ (*p* = 0.252) or MCP-1 (*p* = 0.541) serum levels were also registered in cluster II (Figure 3C).

**Table 5:** Intestinal microbial populations, fecal short-chain fatty acid (SCFA), and serum cytokine levels according to free fatty acids (FFA) clusters.

	Study population (*n* = 101)	Cluster I (*n* = 62)	Cluster II (*n* = 39)	*p*-Value
**Microbial populations (log cells/g)**				
*Akkermansia*	5.84 ± 1.80	6.24 ± 1.84	5.09 ± 1.47	**0.002**
*Bacteroides* group	9.15 ± 1.16	9.05 ± 1.21	9.19 ± 1.17	0.587
*Bifidobacterium*	8.26 ± 0.91	8.02 ± 1.44	8.35 ± 0.95	0.233
*Clostridium* cluster XIVa	8.52 ± 1.41	8.30 ± 1.52	8.63 ± 1.37	0.306
*Lactobacillus* group	6.14 ± 1.04	5.92 ± 1.14	6.41 ± 0.86	**0.024**
*Faecalibacterium*	8.05 ± 1.29	7.84 ± 1.15	8.24 ± 1.11	0.105
**SCFA (mM)**				
Acetate	40.46 ± 19.20	35.43 ± 14.32	49.31 ± 22.98	**0.001**
Propionate	15.04 ± 8.34	13.03 ± 6.01	18.26 ± 10.45	**0.003**
Butyrate	10.61 ± 7.19	9.74 ± 7.07	12.08 ± 7.31	0.135
Total SCFA	66.42 ± 31.06	58.21 ± 23.75	79.66 ± 36.95	**0.004**
**Cytokine levels (pg/ml)**				
IL-6	442.79 (949.99)	411.72 (636.85)	752.40 (1,155.22)	**0.032**
MCP-1	267.30 (127.83)	268.48 (101.13)	263.20 (128.51)	0.541
IFNγ	2.05 (4.70)	2.05 (4.64)	2.48 (4.12)	0.252

*Microbial populations and SCFA fecal levels and serum cytokines from the whole population (*n* = 101) and from the study participants classified according to clusters computed from individual FFA levels are shown. Differences in microbial populations, fecal SCFA, and serum cytokine levels between clusters were analyzed by *t* or Mann–Whitney *U* tests. The *p*-values highlighted in bold represent statistically significant differences. Variables are expressed as mean ± SD or median (interquartile range)*.

Finally, some statistical analyses performed independently within each cluster revealed particular interesting features for each group, thereby stressing the existence of important differences between groups. On the one hand, IL-6 serum levels were negatively correlated to *Akkermansia* abundance (*r* = −0.312, *p* = 0.034) and acetate (*r* = −0.344, *p* = 0.014) in subjects included in cluster I, whereas no associations were observed in cluster II, characterized by higher IL-6 and acetate levels. Conversely, acetate and total fecal SCFA were strongly associated with higher total FFA serum levels in subjects included in cluster II (*r* = 0.459, *p* = 0.006; and *r* = 0.410, *p* = 0.016, respectively). Similarly, total energy intake was positively associated in this group with total SCFA production (*r* = 0.438, *p* = 0.008), but not in their cluster I counterparts (*r* = −0.044, *p* = 0.748), hence supporting a cross talk between host and microbial metabolisms, in which SCFA are involved.

Overall, our findings support the existence of homeostatic relationship between microbial populations, host lipid metabolism and inflammatory mediators. Thus, imbalanced microbial populations and/or increased fecal SCFA production could be related to an altered lipid metabolism as well as a serum IL-6 shift.

### Analysis of the Associations among Impaired FFA Profile, Subclinical Metabolic Alterations, and Microbial Populations

Then, we addressed in our group of adult subjects, whether an altered gut microbiota composition and/or serum inflammatory mediators may have a negative impact on the host metabolism and if a clinical relevance of these players could be expected.

Although no significant differences in serum levels of glucose, cholesterol or triglycerides were found between groups, individuals within cluster II were more likely to exhibit hyperglycemic (>100 mg/dl) or hypertriglycemic (>150 mg/dl) states than those within cluster I (22/39 vs 22/62, *p* = 0.039 and 7/39 vs 4/62, *p* = 0.070). Consequently, metabolic alterations (either hyperglycemia or hypertriglycemia, as stated in the Methods section) were more prevalent in cluster II (23/39 vs 24/62, *p* = 0.047).

The strength of these associations was further analyzed by multiple logistic regression. By selecting subclinical metabolic alterations as the dependent variable and adjusting the model for confounders (age, gender, BMI, total energy, as well as lipids, carbohydrates, protein, and fiber intakes, all introduced as independent variables), we found that individuals within cluster II were more likely to exhibit metabolic alterations (OR [95% CI], *p*: 3.064 [1.127, 8.330], 0.028). Interestingly, this association remained significant when the microbial groups were entered in the model, appearing *Akkermansia* abundance also associated with metabolic alterations (*p* = 0.029). This highlights the interplay between altered FFA profile, microbial populations and impaired host metabolism.

Moreover, additional observations reinforced the finding commented just above. Since our initial results point to a cross talk between host and microbial metabolism, we further deepen into this idea focusing on possible differential associations between microbial groups or SCFA with nutrient intakes. In subjects with subclinical metabolic alterations, *Lactobacillus* abundance was correlated with carbohydrates intake in both cluster I (*r* = 0.603, *p* = 0.004) and II (*r* = 0.765, *p* < 0.001), but with fiber intakes only in cluster I (*r* = 0.609, *p* = 0.003). No associations were observed in those subjects free of metabolic alterations. On the other hand, the association between total SCFA and total FFA levels mirrored this situation, a positive correlation being found in individuals with metabolic alterations in both cluster I (*r* = 0.524, *p* = 0.018) and II (*r* = 0.432, *p* = 0.045), but not in those individuals without subclinical metabolic alterations.

In sum, these results support an association between microbial populations, nutritional factors, and impaired host metabolism. Subjects exhibiting an altered FFA profile, which also display imbalanced *Akkermansia* and *Lactobacillus* populations, were more frequently associated with subclinical pathogenic metabolic states. Divergent patterns in the associations between dietary intakes with microbial populations and SCFA were also observed.

### Microbial Imbalanced Populations As Predictors of Impaired FFA Levels

Finally, although *Akkermansia* abundance was identified as the only microbial predictor of serum FFA levels in the whole population analyzed (*n* = 101), striking differences in *Akkermansia* levels were evidenced between clusters I and II. Since the results commented just above suggest that altered microbial populations and their interactions with nutrient intakes may underlie impairment in the host metabolism and that SCFA can have relevance in such interactions, we included all these parameters (microbial populations, SCFA, dietary intakes, age, gender, and BMI) as independent variables in a multivariate regression analysis, FFA levels being selected as the dependent variable, after stratifying our populations by the clusters obtained in the previous analysis.

Interestingly, whereas only age (*p* = 0.038) and BMI (*p* = 0.050) were identified as predictors of FFA levels in individuals within cluster I (*R*^2^ (model) = 0.407), *Lactobacillus* abundance (*p* = 0.032), fiber intake (*p* = 0.021), SCFA (acetate *p* = 0.008, propionate *p* = 0.018, and butyrate *p* = 0.003), and gender (*p* = 0.008) were associated with FFA levels in subjects within cluster II (*R*^2^ (model) = 0.646).

In summary, different factors were found to be associated with total FFA levels depending on the levels of *Akkermansia*. Above a threshold of *Akkermansia* levels, anthropometric factors such as BMI and age were the main predictors of serum FFA. However, when *Akkermansia* abundance is low, factors other than the anthropometric characteristics (as imbalanced microbial populations or SCFA production) seem to impact the FFA pool.

## Discussion

Despite the important research advances in recent years, the links between human metabolism and gut microbiota are far from being completely understood, especially in the field concerning the lipid metabolism. The present study addresses a multilevel analysis of this scenario, by assessing different surrogate biomarkers of the lipid metabolism, in addition to some relevant intestinal microbial groups and SCFA production as well as inflammatory mediators; the study was performed in a sample of adult subjects from the general population in order to gain some insight into the relationship among these parameters and their potential impact on the human health. Our results revealed an association between the abundance of the intestinal microorganism *Akkermansia* and circulating FFA. This association was restricted to a specific group of FFA species, *Akkermansia* and serum IL-6. In addition, cluster analyses revealed that imbalanced intestinal microbial groups and levels of SCFA production may be related to an impaired FFA profile, pro-inflammatory and saturated fatty acids mainly hallmarking this group. Interestingly, individuals who exhibit these features were more likely to show metabolic alterations. Therefore, the results herein presented provide valuable information on the gut microbiota-host metabolism axis and its involvement in human health.

A major finding of our study was the association between *Akkermansia* and serum FFA levels. *Akkermansia* has been reported to participate in the maintenance of gut integrity and energy harvest by the host (34). It has been confirmed that *Akkermansia* has a causative role in lowering body fat mass and in glucose homeostasis in mice models (35, 36), although evidence in humans is limited. Our results demonstrate for the first time an association between *Akkermansia* and serum FFA in a cohort of human adults from the general population. Interestingly, we were able to cluster these healthy adults into two independent groups on the basis of serum individual FFA species, one of these groups showing an increased prevalence of subclinical metabolic alterations. In our study *Akkermansia* was negatively associated mainly with saturated FFA, in line with the differences that have been previously reported by other authors between lard-like and fish oil-enriched diets in the gut microbiota of mice (36, 37). Moreover, differences in *Akkermansia* abundance found in our study between FFA clusters were associated in turn with striking differences in total FFA levels. This observation supports a gradual relationship between *Akkermansia* abundance and FFA serum levels and therefore aligns with the concept proposed by other authors (36, 38) that a “threshold” for *Akkermansia* levels may exist. In line with this hypothesis, below certain levels of *Akkermansia* abundance, gut barrier, and other functions developed by this microbe may become insufficient, thus promoting a shift from a healthy toward a pathologic-prone status. As previously commented, different parameters were found to be predictors of total FFA serum levels depending on the *Akkermansia* levels. Therefore, low *Akkermansia* levels may render the host metabolism more sensitive to a number of factors which can lead to an imbalanced FFA profile and, potentially, altered metabolism, hence supporting this notion.

*Akkermansia* is known to reside within the mucus layer of the intestine, thus contributing to strengthen the intestinal wall (34). Then, it is feasible that decreased *Akkermansia* levels may lead to a compromised barrier function and increased gut permeability, hence promoting metabolic endotoxemia, which has been related to the development of obesity and associated disorders (35, 39). Interestingly, we have found that decreased *Akkermansia* levels were associated with elevated IL-6 serum concentrations and impaired FFA profile, and the subpopulation with such profile exhibited a bias toward the enrichment in pro-inflammatory and saturated fatty acids, as well as an increased prevalence of metabolic disturbances, thus supporting those previous findings. Therefore, these results suggest that *Akkermansia* may be linked to the inflammatory milieu by modulating the FFA profile in the host.

Previous studies have revealed reduced levels of *Akkermansia* in patients suffering from inflammatory bowel disease or other metabolic impaired conditions (40, 41), although some controversy exist on the role of *Akkermansia* in these disorders. Interestingly, a recent study reported decreased counts of *Akkermansia* in pre-diabetic individuals (42), suggesting an early involvement of this microorganism in metabolic disorders. Thus, we decided to focus on subclinical metabolic features (suggestive of an impaired metabolism), which can be detected in healthy subjects, in order to improve our knowledge on the potential role of *Akkermansia* as an early marker of impaired metabolic conditions in the general population. These metabolic features were related to an increased risk of metabolic complications in the long-term (43–48). Thus, these subjects showing such subclinical alterations could be classified as “at risk” population according to the scientific literature. Based on this approach, it may be hypothesized that imbalanced microbial populations can underlie the subclinical stage of some metabolic conditions. Actually, the lack of association in our study between *Akkermansia* and other clinical hard end-points, such as obesity or related immune mediators, such as MCP-1 or IFNγ, may support a very early role of these features. However, long-term studies are warranted.

Importantly, we found an opposite behavioral pattern between *Akkermansia* and *Lactobacillus* groups, the latter being over-represented in subjects with impaired FFA profile and increased prevalence of metabolic traits. Although classically regarded as “beneficial” microbes, some recent evidence highlights a positive association between *Lactobacillus* and BMI (49, 50) as well as with some pathological outcomes (51–53). In fact, a slight increase in the relative abundance of *Lactobacillales* has been reported in the context of the inflammatory condition of Behçet syndrome (54), related to an altered SCFA production and aberrant immune responses. Thus, these pieces of evidence point to a *Lactobacillus* within-group heterogeneity with relevance for the human health, as suggested by other authors (40, 55). In agreement with this hypothesis our findings on the interactions between *Lactobacillus* abundance and nutrient parameters, evidenced different association patterns as depending on the metabolic “health” status of the subject. Controversy observed in mice studies and clinical interventions in humans is also consistent with this idea. A recent work shows opposite patterns upon mucin usage as substrate by *Lactobacillus* and *Akkermansia* (56), thereby suggesting that trophic interactions may underlie, at least in part, these opposite trends of both microorganisms. Actually, a negative effect on *Akkermansia* levels in the gut upon administration of a probiotic mixture was observed when *Lactobacillus*, but not other probiotic bacteria, were added to the mixture (57). Conversely, *Akkermansia* has been shown to increase the production of the antimicrobial peptide RegIIIγ by colonic epithelial cells (35). This peptide specifically targets Gram-positive bacteria, thus potentially accounting for the opposite trends between *Akkermansia* and *Lactobacillus* groups. In sum, it is feasible that *Akkermansia* may modulate the gut environment and some intestinal microbial populations through several mechanisms (58).

It has been reported that *Akkermansia* and *Lactobacillus* exhibited diverging trends in twins discordant for metabolic syndrome (40). Our results herein presented are in line with these findings and suggest that the altered composition of the intestinal microbiota may be found in subclinical stages in healthy subjects. A recent study in mice revealed that whereas the colonization by *Akkermansia muciniphila* shifted the intestinal mucosa gene expression profile toward gene pathways involved in immune tolerance and metabolic homeostasis, colonization by *Lactobacillus plantarum* resulted in an overexpression of genes involved in the metabolism of fatty acids, lipoprotein lipase being one of the most up-regulated genes (59). An enhanced enzymatic activity of this enzyme may contribute to explain the striking increase in serum FFA found in association with increased *Lactobacillus* abundance in our study. Actually, overexpression of lipoprotein lipase gene has been related to fatty acid accumulation and insulin resistance (60, 61). On the other hand, the balanced immune responses promoted upon *Akkermansia* colonization may also account for the increased IL-6 levels found in our study in subjects with diminished abundance of *Akkermansia*. This is also in accordance with the positive effect on the induction of regulatory T cells reported in mice administered *Akkermansia* (62). It must be noted that IL-6 can promote a number of pleiotropic functions other than triggering inflammation. However, since we have focused our analysis in subjects with no previous diagnosis of chronic or immune-mediated conditions, our approach allowed us to gain insight into the relationships between *Akkermansia*, IL-6 and low-grade inflammation in healthy states. Nevertheless, these associations cannot be directly translated into pathological frameworks. Therefore, a study of the gut microbiota composition—IL-6 axis in disease stages remains to be elucidated.

Modulation by *Akkermansia* of genes involved in lipid metabolism seems to be mediated, at least in part, by the production of SCFA (63). Imbalanced microbial populations can have an impact on SCFA production, which can elicit different responses in the host metabolism and immune system (64). Interestingly, several authors have found increased SCFA in obesity and metabolic syndrome [reviewed in Ref. (65)], acetate and propionate being especially relevant in such alterations (66, 67). The associations found in our study between acetate and IL-6 and that of the total SCFA with FFA serum levels strongly support the cross talk between gut microbiota, host metabolism and immune networks, highlighting a role for SCFA as important elements of this interplay.

The present study has a number of limitations that can be remarked. On the one hand, we have performed a targeted analysis of the gut microbial composition instead of a global profiling by 16S rRNA gene sequences analysis. As a consequence, whether a reduced diversity underlies the present findings cannot be concluded. Similarly, a number of selected FFA and the major SCFA species were chosen for the analysis, based on their outmost relevance in several biological processes. In addition, the same concerns apply to the analysis of inflammatory mediators. Finally, a more precise characterization of dietary intakes may be needed to precisely account for the exact contribution of short-term nutrition to the FFA levels.

**Figure 4:**
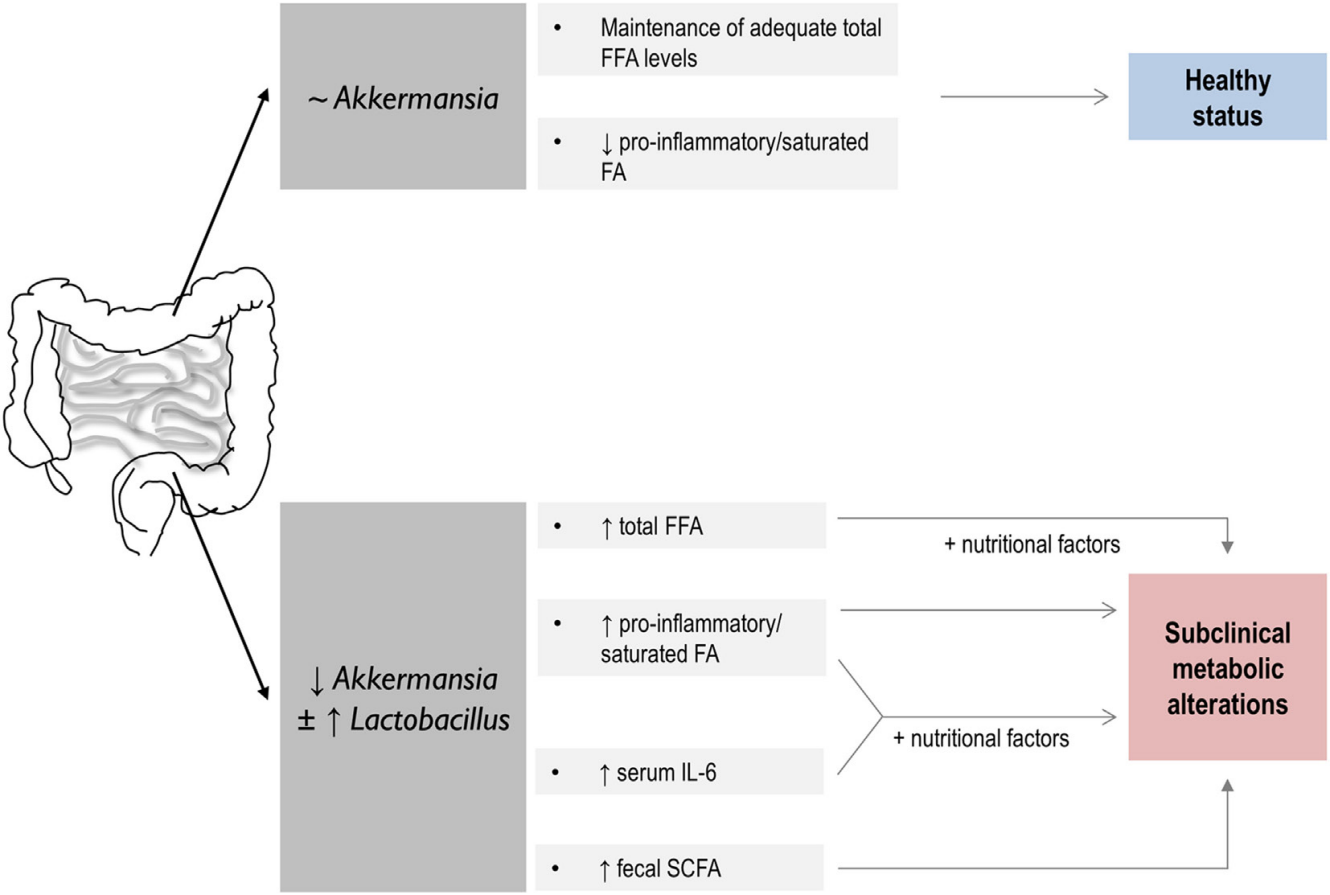
Summary of the global findings of the present study and proposed hypothesis on their potential impact in human health. A link between host lipid metabolism and gut microbiota with potential consequences for the human health is disclosed. On the one hand, above a certain value, *Akkermansia* levels seem to play a role in the maintenance of the serum free fatty acids (FFA) pool in healthy individuals. Decreased *Akkermansia* abundance in the gut microbiota, which seems to coincide in our study with increased *Lactobacillus* counts, is associated with an altered serum FFA profile, hallmarked by striking quantitative and qualitative differences in FFA as well as in IL-6 serum levels and intestinal short-chain fatty acid (SCFA) production. These features, and their interaction with nutritional factors, were related to an increased prevalence of subclinical metabolic alterations. Thus, a link between dysbalanced microbial populations, host lipid FFA metabolism, and human health may be suggested, SCFA production playing an important role.

In conclusion, we reported an association between some intestinal microbial populations and the characteristics of the FFA profile in healthy middle-aged subject. *Akkermansia* and *Lactobacillus* groups seem to be connected to the health metabolic status of the host, the interplay with nutritional parameters playing a potential role (Figure 4). Finally, our findings provide some evidence on the role of SCFA as mediators of the cross talk in the gut microbiota-host lipid metabolism axis. Although our results point to a very early role of an altered microbial composition in the further development of metabolic disorders, prospective and long-term studies are needed to accurately address this possibility.

## Author Contributions

All the authors listed made substantial contributions to the design of the work, analysis, or interpretation of the results obtained; participated in the study design and data interpretation, reviewed the manuscript, and approved the final version; and agreed to be accountable for all aspects of the work in ensuring that questions related to the accuracy or integrity of any part of the work are appropriately investigated and resolved. JR-C and NS performed most of the experimental procedures. SG was involved in the nutritional assessments, anthropometrical measurements, and collection of samples. AM, MG, CR-G, and AS provided biological samples and financial support. JR-C and AS drafted the manuscript.

## Conflict of Interest Statement

The authors declared no potential competing financial interests concerning this study. Funders had no role in study conception, design, analysis of the results, or decision to publish.
